# The Positive Side of the Alzheimer’s Disease Amyloid Cross-Interactions: The Case of the Aβ 1-42 Peptide with Tau, TTR, CysC, and ApoA1

**DOI:** 10.3390/molecules25102439

**Published:** 2020-05-23

**Authors:** Lidia Ciccone, Chenghui Shi, Davide di Lorenzo, Anne-Cécile Van Baelen, Nicolo Tonali

**Affiliations:** 1 Department of Pharmacy, University of Pisa, via Bonanno 6, 56126 Pisa, Italy; 2 CNRS, BioCIS, Université Paris-Saclay, rue Jean-Baptiste Clément 5, 92290 Châtenay-Malabry, France; chenghui.shi@universite-paris-saclay.fr (C.S.); d.dilorenzo94@gmail.com (D.d.L.); 3 Département Médicaments et Technologies pour la Santé (DMTS), CEA, INRAE, Université Paris Saclay, SIMoS, 91191 Gif-sur-Yvette, France; anne-cecile.van-baelen@u-psud.fr

**Keywords:** Alzheimer’s disease, cross-interaction, amyloidosis, TTR, CysC, ApoA1, Tau, Aβ 1-42, peptidomimetic inhibitors, foldamers

## Abstract

Alzheimer’s disease (AD) represents a progressive amyloidogenic disorder whose advancement is widely recognized to be connected to amyloid-β peptides and Tau aggregation. However, several other processes likely contribute to the development of AD and some of them might be related to protein-protein interactions. Amyloid aggregates usually contain not only single type of amyloid protein, but also other type of proteins and this phenomenon can be rationally explained by the process of protein cross-seeding and co-assembly. Amyloid cross-interaction is ubiquitous in amyloid fibril formation and so a better knowledge of the amyloid interactome could help to further understand the mechanisms of amyloid related diseases. In this review, we discuss about the cross-interactions of amyloid-β peptides, and in particular Aβ1-42, with other amyloids, which have been presented either as integrated part of Aβ neurotoxicity process (such as Tau) or conversely with a preventive role in AD pathogenesis by directly binding to Aβ (such as transthyretin, cystatin C and apolipoprotein A1). Particularly, we will focus on all the possible therapeutic strategies aiming to rescue the Aβ toxicity by taking inspiration from these protein-protein interactions.

## 1. Introduction

Over the last decades, more than forty severe degenerative disorders have been added to a group of pathologies called amyloidosis. All of them are characterized by the aggregation of misfolded proteins which have been found to adopt the same amyloid β-sheet-rich architecture, as part of their nature [1,2]. Amyloid fibril formation is generally associated to a protein misfolding, followed by an aggregation process which continues until the formation of insoluble aggregates. The amyloid form of these aggregates can be defined through in vitro observations by cross-β X-ray diffraction pattern while their structure can be observed by transmission electron microscopy (TEM) or atomic force microscopy (AFM). The formation of their ordered molecular structure can be also revealed by fluorescence spectroscopy with thioflavine T and Congo red dyes [3].

Alzheimer’s disease (AD) belongs to this group of amyloidosis. AD is a progressive neurodegenerative disorder associated with cognitive decline and is considered the most common form of dementia in the elderly [4]. In amyloid plaques, in the gray matter of the brain, the two classical lesions are the depositions of intracellular neurofibrillary tau tangles and the extracellular deposits of aggregated amyloid-β (Aβ) peptides [5]. Nowadays, it is widely recognized that an imbalance between production and clearance of Aβ peptides in the brain results in accumulation and aggregation of Aβ. Aggregates of toxic Aβ in the form of soluble Aβ oligomers, intraneuronal Aβ, and amyloid plaques injure the synapses and ultimately cause neurodegeneration and dementia [6,7].

One of the strategies adopted to stop or reverse the progression of the disease is to modulate or inhibit the aggregation process of Aβ, by various mechanisms: stabilization of its native state, destabilization of its incorrectly folded state [8], bypass of the on-pathway oligomer formation, inhibition of the fibril elongation and disaggregation of the already formed amyloid aggregates [9,10,11,12].

Several natural polyphenols have been reported to exhibit potent inhibitory action against Aβ aggregation [13,14]. In parallel, research in this field was also oriented towards peptides which can be classified in two different major groups. The first class is composed by peptides that are similar in sequence to wild type proteins and they are termed as rationally designed peptides. Instead, the second class is characterized by peptides which are identified from libraries, that may or may not show sequence similarly to wild type, and these are termed as randomly generated peptides. Other approaches have been exploited in the field of peptidomimetics, such as synthetic peptide derivates-β sheet breakers and β peptide hairpins [9,10,11,12]. 

Overall impairment in Aβ clearance is also a major contributor to disease development [13]. Molecular chaperones represent the most important elements of the ensemble of machinery responsible for protein homeostasis [14]. For example, apolipoprotein E (Apo-E), the major cholesterol carrier, has an important role in modulating Aβ metabolism, aggregation and deposition [15]. Depending on the APOE polymorphic alleles, Apo-E isoforms exhibit differential lipidation status, which affects Aβ clearance in an isoform-dependent manner. Alternatively, Apo-E may sequester Aβ and promote cellular uptake and degradation of Apo-E-Aβ complexes [16]. In addition, Apo-E might modulate Aβ removal from the brain to the systemic circulation by transporting Aβ across the blood-brain barrier [17]. The exact mechanism by which Apo-E isoforms differentially regulate Aβ aggregation and deposition requires further investigation. More recently, it has been shown that a homozygous APOE3ch mutation can impart resistant to the clinical onset of AD pathogenesis, probably having beneficial effects on downstream tau pathology and neurodegeneration, even in the face of high Aβ plaque burden [18]. Therefore, inquiring the role of this chaperone can enhance the understanding of the Aβ misfolding-dependent aggregation process and allows to develop alternative therapeutic strategies to treat AD. In 2013, the currently being explored approaches are well resumed and discussed in a review published by Liu et al. [19]

Several other factors seem to contribute to the development of AD, thus questioning the amyloid cascade hypothesis and revealing its complex process linked by multiple interconnected events that cannot be easily explained by a single hypothesis. Among these factors, we could count lysosomal disfunction, loss of Ca^2+^ homeostasis, neuroinflammation, progressive oxidative damage and problems related to glucose metabolism [20,21]. All of them represent the pathogenic steps or pathways of the disease and targeting or altering them might be prevent the progression of the disease. 

However, although AD progression is widely recognized to be connected to Aβ1-42 aggregation, several other processes likely contribute to the development of AD and some of them are related to protein-protein interactions. These latter are the quintessence of physiological activities, but also participate in pathological conditions. Amyloid formation can be considered an abnormal protein-protein interaction process [22]. The progression of AD implicates more than one protein and this, together with the synergistic occurrence between amyloid proteins (cross-interaction) [23], allows to study the disease with another point of view, giving the opportunity to explore novel therapeutic approaches. The strength of better understanding the amyloid interactome lies in the perspective to identify key mediators of amyloidogenicity or key interactions with other amyloid proteins that could be targeted therapeutically. 

## 2. Amyloid Cross-Interactions

Amyloid aggregates usually contain not only single type of amyloid protein, but also other types of proteins. Some studies revealed that Aβ is just the major amyloid protein of the 488 proteins in AD related amyloid plaque [24]. In Parkinson’s disease (PD)-related Lewy body, around 550 proteins were detected, including α-synuclein, synphilin-1, tau and many others [25,26,27].This phenomenon can be rationally explained by the process of protein cross-seeding and co-assembly. Moreover, several amyloid proteins show the abilities to affect the aggregation of other amyloid proteins. For example, islet amyloid polypeptide (IAPP) was reported to promote the α-synuclein amyloid formation, which can explain why type-2 diabetes patients are susceptible to developing PD [28]. Some experiments suggested that tau and α-synuclein can influence each other, accelerating their respective fibrillization process and resulting in the formation of pathological inclusions in neurodegenerative diseases [29]. All these evidences indicated that amyloid cross-interaction is ubiquitous in amyloid fibril formation.

We can divide the amyloid cross-interaction into two categories based on their effect to progress of diseases. One is amyloid cross-interaction with positive effect, another one is amyloid cross-interaction with negative effect. The positive effects include inhibiting the formation of amyloid oligomers or amyloid fibrils, reducing the toxicity of aggregates, promoting the degradation of aggregates and promoting the dissociation of aggregates. In contrast, the negative effects include promoting the aggregations, increasing the toxicity of aggregates and inhibiting the degradation of aggregates. These effects are not mutually exclusive. Sometimes one amyloid protein which inhibits the amyloid fibril formation of another amyloid protein, simultaneously reduces the toxicity of the aggregate, like the effects that transthyretin (TTR) has on Aβ1-42 [30]. Thus, the amyloid proteins interactome is usually complex and can involve diversified cross-interactions between one protein and different other proteins. To date, many amyloid cross-interactions have been attracted attention and a better knowledge of them could be helpful for further understand the pathological mechanisms behind the amyloid related diseases. 

As mentioned above, AD related amyloid plaque contains more than 400 proteins, including Aβ, Tau, cystatin C, IAPP, α-synuclein, TTR, etc. Moreover, a great many studies revealed that lots of amyloid proteins can cross-interact with Aβ. For example, there are synergistic amyloid cross-interactions of Aβ and α-synuclein that promote mutually aggregations not only in vitro but also in vivo [31,32,33]. The Tau protein, a major constituent of neurofibrillary tangle, also shows the mutual influences with Aβ on aggregation and toxicity [34]. These cross-interactions between Aβ and other amyloid proteins may play a critical role in AD progression. They provide a new strategy to design novel molecules that mimic the cross-interaction with Aβ, although the mechanisms of these interactions have not been completely figured out.

**Figure 1:**
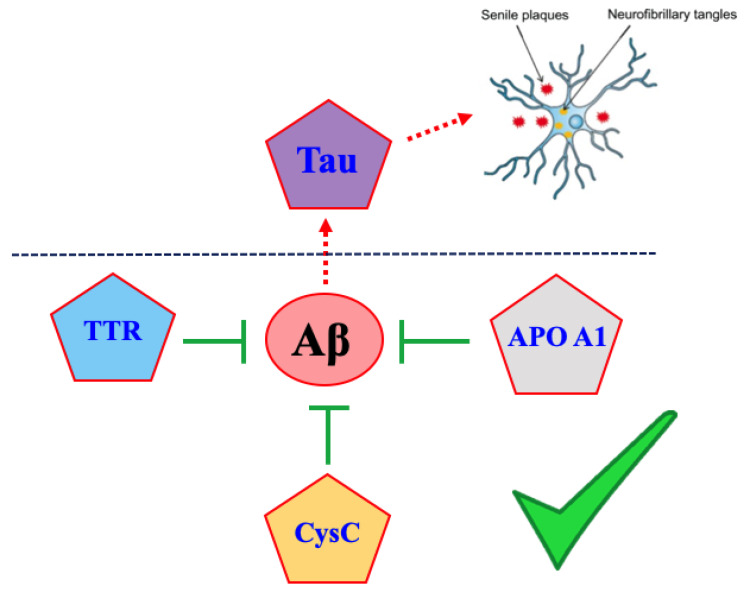
A cross-amyloid network between Aβ peptide and four amyloidogenic proteins. Proteins with intrinsic amyloidogenic potential are contoured by red lines. Green symbols: amyloid proteins that have a positive effect against the progression of AD. Red dashes arrow: amyloid protein pathway according to the amyloid cascade hypothesis. The details of the interactions are discussed in the review for each protein.

In this review, we discuss about the cross-interactions of Aβ1-42 with other amyloids, which have been presented either as integrated part of Aβ1-42 neurotoxicity process (such as Tau) or conversely with a preventive role in AD pathogenesis by directly binding to Aβ (such as transthyretin, cystatin C and apolipoprotein A1), Figure 1. Particularly, we will focus on all the possible therapeutic strategies set up until now with the aim to rescue the Aβ1-42 toxicity by taking inspiration from these protein-protein interactions. 

## 3. Amyloid Proteins Displaying Cross-Interaction with Aβ1-42 Peptide

### 3.1. Tau Protein

Histologically AD is characterized by extracellular senile plaques of amyloid β (Aβ) and intracellular neurofibrillary tangles of hyperphosphorylated Tau (NFT) [35,36,37]. Evidences suggest that senile plaques deposits do not correlate well with the progression of the cognitive decline, whereas Tau aggregation seems to do. In fact, higher is the amount of Tau aggregates, greater it seems to be the cognitive impairment and the severity of symptoms [6,38,39,40]. 

Human Tau protein is located in neurons, where it participates to the axonal stability by interacting with tubulin, promoting its assembly into microtubules and reinforcing pre-established microtubule structures. It is encoded by a single gene, MAPT, on chromosome 17 containing 16 exons [41]. The major form in the human brain is encoded by 11 exons. Exons 2, 3, and 10 are alternatively spliced and the transcription leads to the formation of six Tau isoforms [42,43], displaying three or four microtubule binding repeats (3R or 4R) if exon 10 is respectively absent or present [44]. Tau is considered as an intrinsically disordered protein (IDP) because, in solution, a variety of spectroscopic techniques including circular dichroism (CD), nuclear magnetic resonance (NMR) and Fourier transform infrared (FTIR) spectroscopy have shown that Tau is lacking of a secondary and tertiary structure [45,46,47,48]. Tau’s modulation of tubulin assembly and stability is regulated by its degree of phosphorylation. In pathological conditions, such as Alzheimer’s disease (AD), Tau protein undergoes a hyperphosphorylation, which leads to its conformational transition into β-sheet rich structures and thus its self-assembly into large and insoluble tangles [49,50,51]. Neurofibrillary lesions are made of paired helical and straight Tau filaments (PHFs and SFs), whose structures have recently been elucidated in high-resolution through cryo-electron microscopy (EM) [52]. 

For several years, Aβ extracellular aggregation and Tau intracellular deposition were thought to be two separate hallmarks of AD and it was widely accepted that the pathogenesis of the disease could be related to only one of these two events. More recently, with the advance of knowledge and studies in this field, more and more evidences revealed that Tau and Aβ are strictly and mutually interconnected in AD pathogenesis [53]. It is still debating how the aggregation of one of these two amyloid proteins could affect the other. Genetic data, as well as autopsy and neuroimaging studies in patients with AD, indicate that Aβ plaque deposition precedes cortical Tau pathology [54] and that the accumulation of Aβ exacerbates Tau’s pathology [6,55,56]. Others, instead, support the idea that it is mainly Tau that plays the major role in the etiopathology, essentially for two reasons: firstly because Tau tangles can be found in patients’ brain even when extracellular Aβ deposits are not present [57], and secondly because evidences proved that intracellular Tau tangles rather than Aβ deposits are the most neurotoxic species, mainly responsible of the serious neurotoxic effects, behavioral deficits and cognitive decline associated with the progression of the disease [38,58,59]. The observation of co-localization of oligomeric Aβ and phosphorylated Tau in AD brain patients led some research groups to think that a mutual cross-interaction between the two amyloid proteins might be responsible of the pathological behavior of both proteins [60]. It is the formation of an Aβ-Tau-complex that could prime the Aβ nucleation and the Tau hyperphosphorylation [34]. A great number of works concerning Aβ and Tau together, both in vitro and in vivo, supports the cross-seeding theory [61,62] or, better, the interaction and the synergistic effects [63] of these misfolded proteins. Despite this, the mechanism by which one influences the other is still not clear, and several questions arise. To date, three main suggested mechanism have been hypothesized: (1) Aβ species seem to interacts with kinases, thus enhancing the phosphorylation of Tau and its detaching from microtubule with consequently aggregation [64,65,66,67,68], (2) the Aβ-induced Tau phosphorylation is mediated by soluble inflammatory factors from astrocytes [69] (3) Aβ seeds and propagates Tau’s aggregation through a direct interaction with it [60,70,71]. In this review regarding amyloid cross-interactions, we decided to mainly focus on the direct interaction between Aβ and Tau, highlighting what is emerged in the last years. Since a very recent and detailed review about the in vivo intracerebral seeding of Aβ and Tau in mice [72], has just been released, we will mainly focus on the in vitro studies aiming to deepen the knowledge about the physicochemical aspects of this interaction. The growing interest in understanding the cross-seeded interaction between Aβ and Tau is justified by several in vivo experiments showing that Aβ enhances Tau pathology by increasing the formation of Tau species capable of seeding new aggregates [73,74,75,76,77,78,79,80].

In the study of Vasconcelos et al., it has been shown that pre-aggregated Aβ can directly induce Tau fibrillization by cross-seeding in a cell-free assay and that Aβ-seeds can cross-seed Tau pathology and strongly catalyze pre-existing Tau-aggregation in a cellular Tau-aggregation experiment. All these results were successively confirmed by in vivo experiments and revealed the propagating potential of heterotopic seeding of filamentous Tau-aggregates induced by Aβ along functionally connected brain regions [77,79,81].

Immunostaining studies performed by Imamura et al. showed higher co-localized accumulation of toxic Aβ1-42 oligomers and hyper-phosphorylated Tau protein (p-Tau) in hippocampal and cortical neurons, indicating their co-aggregation. The formation of toxic Aβ1-42 oligomers and its co-aggregation with p-Tau oligomers was attributed to insulin deficiency. This in vivo study conducted on AD mouse model proved that the attenuation of insulin signaling is involved in an increase of toxic Aβ1-42 conformer levels which promotes not only an increase in p-Tau but also a direct interaction between the two misfolded proteins with the formation of their co-aggregates [67].

Not all types of Aβ aggregates promote Tau aggregation in the same way. Transduction of Aβ oligomers into the cells enhances more the Tau-aggregation than Aβ-fibrils [82]. Interestingly, the transduction of the cells with Aβ oligomers have no effect on α-synuclein seeding, suggesting that the seeding enhancement by Aβ oligomers is specific to Tau. The mechanism behind this interaction is currently unknown but the hypothesis of a channels/pores formation induced by Aβ oligomers does not seem plausible because these latter should appear in a shorter amount of time (1 to 2 h) [83] respect to the incubation time needed to prime cells and enhance Tau-aggregation. Neither the common cross-seeding hypothesis seems to explain the Aβ-induced Tau aggregation because none of the Aβ-seeds were able to induce Tau aggregation earlier than the usual 24 h of incubation necessary to enhance the Tau-aggregation [84]. 

Guo et al., by using western blot and ELISA experiments, demonstrated the existence of a stable and soluble Aβ-Tau complex able to enhance Tau phosphorylation. It has been suggested that soluble Aβ could bind to soluble non-phosphorylated Tau, promoting then phosphorylation and Aβ accumulation. Peptide membrane arrays showed that Aβ binds to multiple Tau epitopes, especially in exons 7 and 9, and that Tau binds to multiple Aβ peptide sequences in the mid to *C*-terminal regions [34]. Surface plasmon resonance (SPR) analyses showed that Aβ binds to Tau around 1000-fold higher than Tau by itself, suggesting the hypothesis that blocking the sites where Aβ initially binds to Tau might arrest the simultaneous formation of tangles in AD. 

Next to the hypothesis that Aβ influences Tau pathology, Wallin et al. proposed, conversely, a potential interaction mechanism for the influence of Tau on Aβ fibrillation. By nuclear magnetic resonance (NMR), circular dichroism (CD) spectroscopy and photoinduced cross-linking methods, they found that Tau can prevent the in vitro Aβ1-40 fibrillation at stoichiometric Aβ/Tau ratios and to block it at the oligomeric stage. Tau-441 does not induce any conformational change in Aβ monomers and, conversely, prevent the formation of β-sheet rich structure [85].

Thanks to the development of a 3D AD human neuronal cell culture model displaying both extracellular Aβ-deposits (plaques) and the concomitant presence of p-Tau in neurons and fibrillar Tau aggregates like NFT in neurites and cell bodies [86,87], Kwak et al. provided a direct evidence that it exists a direct correlation between Aβ species and Tau pathology in AD [88]. Particularly, the Aβ1-42/1-40 ratio drives the Tau pathology because in the condition of a high Aβ1-42/1-40 ratio more toxic Aβ oligomeric structures are produced. In literature it is known that Aβ1-42/Aβ1-40 mixture rapidly forms small spherical oligomers which are more toxic than oligomeric preparation composed of either Aβ1-40 or Aβ1-42 [89,90,91]. Therefore, as an alternative hypothesis, Aβ1-40 might play a protective role and might counteract Aβ1-42 toxicity. It has been proved for example that transgenic mice expressing high level of Aβ1-40 do not develop Aβ plaque [92] and that Aβ1-40 stabilizes Aβ1-42 monomers by competing for binding site on pre-existing Aβ1-42 aggregates, thus inhibiting further aggregation [93]. Aβ1-40 does not seem to promote Tau phosphorylation but conversely decreases the phosphorylation at Ser262, thus maintaining the binding of Tau to microtubules [94]. It might be interesting, in the future, to develop alternative therapeutic approaches that selectively reduce the Aβ1-42/Aβ1-40 ratio. 

As mentioned above, it is clear that Aβ/Tau amyloid cross-interactions likely contribute to the synaptic dysfunction involved in AD, but much more has to be still performed to better understand the role that each single protein has on the other and which one influences more the pathological behavior of the other. 

The mechanism that triggers Tau aggregation by a direct interaction with Aβ is still a matter of debate and different hypothesis has been proposed in the last years.

Dynamics simulations aiming to understand the mechanism behind the interaction between Aβ1-42 and Tau have been performed by Qi et al. and showed that Aβ oligomer stretches Tau into a more extended conformation by reducing the metastable secondary structures/hydrogen bonding/ salt-bridge networks in Tau monomers and promoting then the exposition of Tau’s fibril nucleating motifs, VQIINK and VQIVYK [71]. Tau’s K18 and K19 constructs interact with both two conserved patches around Tyr10 and Ile41 of Aβ1-42. Particularly, the interaction with residue Ile41 is consistent with experimental observations that Tau pathogenesis is promoted by Aβ1-42 but not Aβ1-40. 

By employing coarse-grained molecular dynamic simulation, the effect of Aβ1-40 fibrils on the aggregation of Tau-RD (Tau’s repeat domains) has been recently investigated. Tau-RDs have high affinity for Aβ1-40 fibrils, and the ^261^GSTENLK^267^ fragment of Tau drives Tau-RD towards the ^16^KLVFFA^21^ fragment of Aβ40 fibrils. The ability of Aβ1-40 fibrils to bind Tau-RD seems to depend on the hydrophobic core fragment of Aβ adopting an extended conformation. Monomeric Aβ1-40, compared to the fibril forms, rarely has this peptide fragment in an extended conformation and this could explain its lower affinity for Tau. They suggest that the different behavior between Aβ1-40 and Aβ1-42 in influencing the Tau aggregation could correlate with the different propensity of these amyloids to aggregate. In that case, the major role of Aβ1-42 in spreading Tau pathology could be ascribed to its greater tendency to self-assembly than Aβ1-40 [95].

Taking inspiration from this cross-interaction between Aβ and Tau, Mohamed et al. decided to study the role of PHF6 fragment of Tau on the Aβ fibrillogenesis. The N-acetylated and C-amidated PHF6 (Ac-VQIVYK-NH_2_) drastically promotes the aggregation of both Aβ1-40 and Aβ1-42 but at the same time it is able to reduce cellular toxicity mediated by Aβ1-40 and Aβ1-42 in hippocampal neuronal cell line (HT22) [96]. By employing molecular docking studies, they observed at the molecular level that PHF6 interacts with the hydrophobic ^14^HQKLVFFA^21^ segment of Aβ in an antiparallel fashion with the Lys undergoing polar interactions with the PHF6 backbone amides. Thanks to this interaction the AcPHF6 hexapeptide can stabilize the β-hairpin structure of Aβ and promote rapid Aβ self-assembly and growth to form less-toxic oligomers or fibrils. 

In a recent study [97] the crystal structure of an Aβ core segment (Aβ16-26) has been determined by Micro Electron Diffraction (ED) and, starting from these results, peptide-based inhibitors of Aβ aggregation have been designed. The Aβ core sequence is implied not only in self aggregation but it seems to be also involved in the cross-seeding interaction with Tau VQIINK and VQIVYK sequences as demonstrated by previous in vitro and in vivo studies [34] and by computational seeding models [98]. These inhibitors proved to be able to reduce the related Aβ toxicity preventing self-aggregation and avoiding Tau cross-seeding by capping Aβ aggregates. All these results open the hypothesis of a pathological cross-seeding via a shared epitope between Aβ and Tau [99]. This study suggest not only that future inhibitors should target common interface region of Aβ and Tau but also that the determination of the high-resolution structure of Aβ-Tau complex would contribute to the understanding of the key binding residues for optimized inhibition of amyloid seeding in AD.

Finally, the cross-interaction between the two proteins could be even more complicated and could require a third protein partner. Gomes et al. found that cellular prion protein (PrPC) may play a role in the progression of AD pathology together with Tau and Aβ. An in vitro pull-down assay confirmed that PrPC is able to interact with Aβ and p-Tau. Co-immunoprecipitation and proximity ligand assay showed an association with Aβ-PrPC and Tau-PrPC both in mice and in human AD brain tissue. PrPC may act as an important mediator of Aβ-driven effects on p-Tau pathology. PrPC behaves as an interaction partner of soluble Aβ oligomers and intervenes in p-Tau propagation by activating, once complexed with Aβ, a signaling pathway that increase the levels of p-Tau [100]. PrPC may provide a novel therapeutic target for stopping p-Tau spreading and its downstream neurodegenerative and cognitive consequences in AD. 

### 3.2. Transthyretin (TTR)

**Figure 2:**
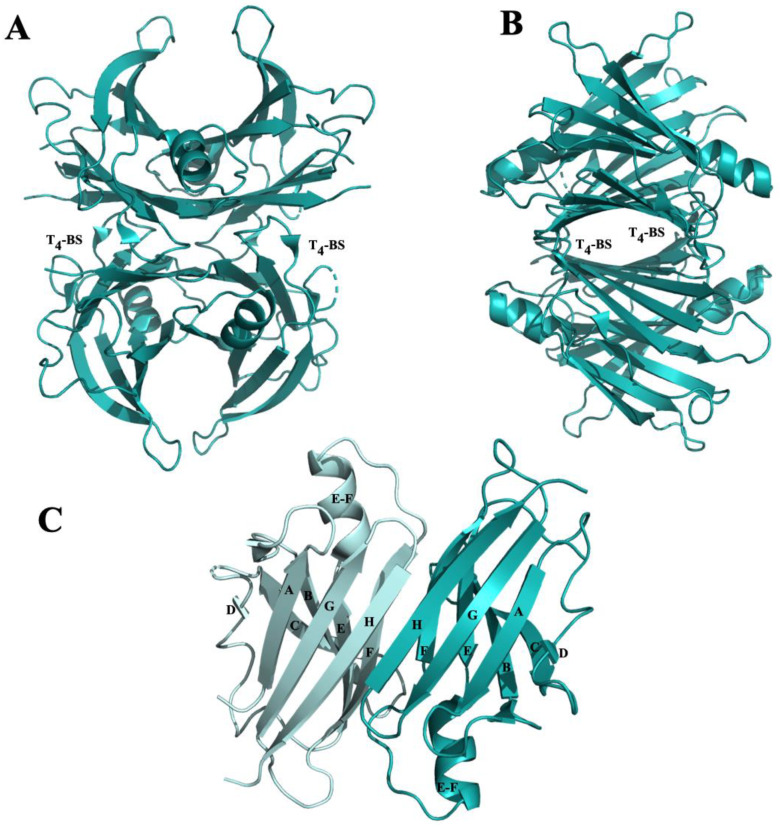
Graphic representation of TTR tetramer. (**A**) The TTR tetramer composed by four equal monomers assembled by 222 molecular symmetry. The tetramer is crossed by thyroxin binding pockets (T4-BP). (**B**) The TTR tetramer rotated of 90°. (**C**) Representation of the dimer composed by two identical monomers. Each monomer is composed by strands D, A, G H, C, B, E and F, and a short EF α-helix.

Human transthyretin is a homo-tetrameric protein characterized by four identical subunits of 14 kDa each. The four monomers, through hydrophobic interactions, are assembled in couples of dimers and two dimers are associated back to back to form a tetramer. The TTR tetramer assembly is characterized by 222 molecular symmetry which forms, in the middle of the tetramer, two identical funnel-shaped named thyroxine binding sites (T4-BS), located at a dimer–dimer interface [101] (Figure 2A,B).

TTR is mainly synthesized by the liver and the choroid plexus of the brain, in minor amounts in the retina [102] and in human placenta [103]. Therefore, it circulates both in human plasma and in the cerebrospinal fluid (CSF), but at different concentrations. The TTR turnover, in the plasma, is relatively rapid with a half-life of approximately two-three days. Under physiological conditions, TTR tetramer transports retinol and thyroxin, as a backup carrier, both in plasma and cerebral spinal fluid [104]. In elderly people, the native TTR tetramer can became unstable favoring the TTR monomeric form which can misfold causing the fibril formation. In aged patients, the fibrils accumulation in organs and tissue induces the onset of senile systemic amyloidosis diseases (SSA) [105,106]. 

TTR tetramer is usually stable, exception when a single point mutation occurs and drastically decreases its stability, thus promoting amyloidosis. Familial amyloid cardiomyopathy (FAC) is a rare autosomal-dominant disease associated to the deposition of TTR amyloid plaques in the myocardium [107] and related to the most common TTR mutation Val122Ile [108]. Familial amyloid polyneuropathy (FAP) is another TTR amyloidosis and is usually associate to Val30Met point mutation [106]. One therapeutic strategy against TTR amyloidosis is the tetramer stabilization by small molecules such as bisaryl [109,110] or monoaryl [111,112,113] structure-based compounds or natural molecules [114,115]. 

In contrast with its intrinsic amyloidogenic potential, TTR can interact with Aβ and play a protective role in AD by sequestering Aβ and reducing protopathic stress. TTR has been described as the major Aβ binding protein in CSF and its interaction with Aβ inhibits the amyloid formation [116]. A direct implication of TTR in AD physiopathology have been confirmed by in vivo studies in AD patients where TTR concentration was observed to decease both in plasma and CSF [117,118]. Moreover, several in vivo experiments, performed in AD transgenic mice, recognized the neuroprotective effect of TTR against Aβ amyloid deposition and toxicity [30,119,120,121,122]. The precise mechanism by which TTR binds to Aβ remains unknown. Several hypotheses have been proposed and controversial results have been obtained. It has been reported that TTR binds to soluble, oligomeric and Aβ fibrils [121,123] performing its relevant role in Aβ clearance, however it is not clear which form of TTR binds to Aβ. Some studies showed that is the TTR monomeric form which binds to Aβ [124,125]. In contrast with this data, in vivo experiments reported that the administration of TTR tetrameric stabilizers to AD transgenic mice led to an improvement of pathological conditions, supporting the hypothesis that it is the TTR tetramer that interacts with Aβ peptide [123]. Recently, ThT fluorescence spectroscopy analyses showed that both TTR tetramer and monomer bind to Aβ1-40 oligomers and inhibit the primary and secondary nucleation processes, which limits both the toxicity of Aβ1-40 oligomers and the ability of the fibrils to proliferate [126]. 

The low-density lipoprotein receptor-related protein 1 (LRP1) is one of the receptors involved in efflux of Aβ across the blood-brain-barrier (BBB). It has been hypothesized that TTR binds to Aβ and this established complex, through the LRP1 receptor, is transported outside of the brain towards the liver [127]. Recently, the same authors reported that the stabilization of the TTR tetrameric structure is essential to allow not only the scavenger of Aβ from the BBB to liver but also the regulation of LRP1 expression and activity [128]. 

In the next two sections we report several studies focused on TTR-Aβ interaction which have been done in the last years. In particular, we discuss the different hypotheses regarding the mechanism by which TTR can bind to Aβ, then we report the state of the art of the therapeutic approaches based on TTR-Aβ interaction which are currently studied against AD.

#### 3.2.1. β-Amyloid-Binding Sites on TTR

Each monomer of TTR contains two four-stranded β-sheets, one “inner” β-sheet of strands D, A, G and H, and another “outer” sheet of strands C, B, E and F, and a short EF α-helix (Figure 2C). 

First analyses of the binding interaction realized by tandem mass spectrometry of cross-linked TTR-Aβ fragments showed that Aβ binds only slowly and relatively weakly to the TTR tetramer, and that the binding is mediated primarily through Aβ aggregates rather than through Aβ monomers. The binding is governed by a hydrophobic interaction between strand A in the inner β-sheet and the amyloidogenic domain on Aβ, region that is sterically restricted in TTR tetramer. A second binding region was identified in the EF helix which is highly solvent exposed and thus less restricted in the TTR tetramer [125]. By using two other complementary methods, or rather SPOT peptide array and single-point mutants, the same research group could affine the previously obtained results and identify strand G and strand E through EF helix/loop as the strongest binding regions of Aβ. Binding to TTR is primarily mediated through two bulky hydrophobic leucine at positions 82 et 110. The slight discrepancy between the two studies is mainly due to the drawback of the cross-linking that it allows to identify only spatially close domains containing lysine [124]. The role of each sequence in the mechanism of binding was successively explored by studying the two L82A and L110A TTR mutants relatively to how they mediate protection against Aβ-induced neuronal toxicity compared to wild type TTR. It was shown that the loss of binding sites reduces TTR protection against Aβ toxicity and that they are the Aβ soluble aggregates that bind preferentially to TTR. By circular dichroism analyses and native gel electrophoresis, it was demonstrated that binding of Aβ could induce a change in wild-type (wt) TTR structure, leading to destabilization of the tetramer. This dissociation might be carried by the first interaction of Aβ with the EF helix/loop region behaving as a sensor of the presence of soluble toxic oligomers. Successively, the dissociation allows to expose the hydrophobic inner sheet (strand G) and to interact with other Aβ peptide. This second interaction might scavenge the toxic oligomers and prevent them from causing cell death [129].

A recent STD-NMR studies conducted on the interaction between TTR and Aβ (12–28) peptide provided a structural model for the TTR-Aβ binding interaction. The central hydrophobic core of Aβ (VFF epitope) is the main structural motif for the recognition and it is able to bind at the surface of the TTR protein, coincident with the surface binding region of EGCG [130], instead of the T4 binding pocket as previously assessed [121].

Buxbaum et al. showed a direct interaction between Aβ (18-21) residues and the thyroxine binding pocket of the TTR tetramer, through nuclear magnetic resonance and epitope mapping by isothermal titration calorimetry (ITC). Their experiments showed a reduced inhibition of Aβ aggregation when the T4 site is occupied by small molecules, confirming the involvement of this site in Aβ binding. In that case, the L82, rather than serving as an Aβ oligomer sensor, may influence the orientation of the side chain of W79, which usually points to the T4 binding pocket [121]. 

In AD patients, the metals ions levels detected in cerebral amyloid plaques drastically grow up and, for example, the total copper concentration could increase up to 400 μM [131]. It has been demonstrated that Aβ peptide is highly sensitive to metal ions such as Zn^2+^, Cu^1+,2+^, Fe^2+,3+^, Mn^2+^. These latter have been shown to have a role in Aβ fibril formation and toxicity, by inducing several conformational changes of Aβ peptide [132,133,134,135]. It has been reported that the same cations interact with TTR [136,137]. In 2018, it has been hypothesized that the TTR-Aβ interaction was modulated by metal ions. Different experiments were performed using bio-layer interferometry (BLI) between TTR and the biotinylated peptide Aβ (1–28) with various CuCl_2_ concentration (0–12.5 mM) and the results showed that the affinity of TTR for Aβ (1–28) is modulated by copper [138]. Moreover, the crystal structures of TTR obtained in presence of Cu^2+^ and Fe^2+^ showed a conformational change comparable to that found for the TTR-rhenium complex in which the distances between L110 and L110’ increased up to 8.5 Å in one T4-BP, while decreased in the other probably due to the rhenium binding [139]. Moreover, the monomer B in asymmetric unit changes its conformation and the E-F helix and residues 85–92 undergo a rearrangement resulting into variation of the dimer-dimer interface. Although the BLI experiments clearly demonstrated that the TTR interaction with Aβ is mediated by Cu^2+^, TTR crystals grown in the presence of CuCl_2_ and Aβ did not show any ordered Aβ peptides.

#### 3.2.2. TTR-Aβ Interaction-Based Strategies to Design Anti-Aβ Agents

**Figure 3:**
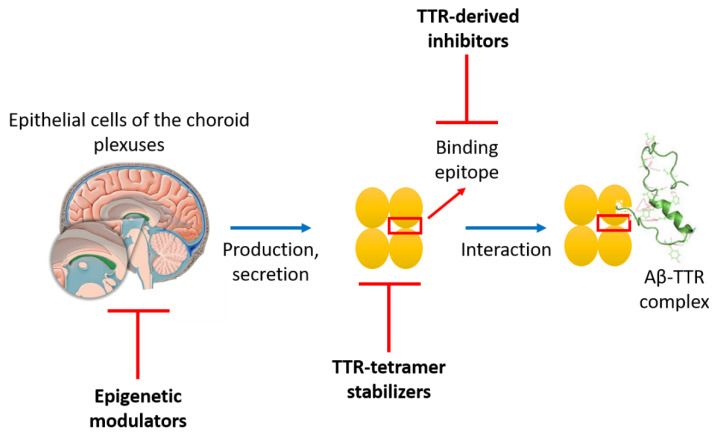
Schematic representation of the three different strategies employed to design anti-Aβ agents based on TTR-Aβ interactions.

Three different strategies have been employed to design anti-Aβ agents based on TTR-Aβ interactions: the epigenetic modulation of TTR, the stabilization of the TTR tetramer and the design of TTR-derived peptide inhibitors (Figure 3). All these strategies have the aim to enhance or mimic the TTR-Aβ interaction in order to improve the clearance of Aβ peptide and consequently avoid its aggregation into amyloid aggregates. 

Quintela et al. demonstrated that sex hormones, such as 5α-dihydrotestosterone, 17β-estradiol and progesterone, increase TTR mRNA and protein level in the choroid plexuses, through ligand activation of hormone receptors which dimerize and interact with specific response elements directly binding to steroid receptor co-factors. This activation cascade promotes the expression of TTR and therefore might have an impact on AD progression. Further studies will be required to establish a clear connection between ovarian hormones, TTR and Aβ degradation [140,141,142]. In a review of 2014 [143], about amyloid-clearing proteins and their epigenetic regulation as a therapeutic target for AD, Turner et al. cited TTR as an amyloid protein with anti-Aβ amyloidogenic effect. TTR could be clearly considered as a transport protein involved in the Aβ clearance mechanisms in the brain whose expression could be regulated to fight again the undesirable accumulation of Aβ toxic aggregates and to prolong Aβ normal functioning. TTR seems to have a similar epigenetic regulation as neprilysin (NEP), an amyloid-degrading peptidase whose expression is regulated by the APP intracellular domain (AICD) and clearance by the histone deacetylase (HDAC). Consequently, inhibitors of HDAC might have the advantage to up-regulate TTR expression in the brain [143].

Ribeiro et al. initiated in 2014 the exploration of iododiflunisal (IDIF), a TTR-tetramer stabilizer, as a new therapeutic approach, aiming to stabilize the tetramer conformation of TTR to promote its binding to Aβ and consequently its clearance. In a first attempt, they studied the effect of an oral administration of IDIF in transgenic mice and they observed the ability of IDIF to bind TTR in plasma and stabilize the protein until entering the brain. Once in the brain, IDIF resulted not only in a decreased brain Aβ level and deposition but also in improved cognitive function. This was the first in vivo evidence that a TTR-stabilizer might be used as a therapeutic agent for AD [122]. Successively, starting from these results, the research group continued to go deeply insight by exploring the biodistribution features of IDIF by radiolabeled techniques [144], the thermodynamic characteristics of the formation of binary (Aβ/TTR) and trinary (Aβ/TTR/IDIF) complexes by calorimetric studies in comparison with tafamidis and diflunisal [145] and the structural features of the interaction by STD-NMR spectroscopy methods [130]. In a different work, administration of resveratrol in mouse model also produced decreased brain Aβ burden and raised plasma TTR concentration, even if the authors revealed that TTR liver gene transcription was not altered. They hypothesized that the instability of TTR tetramer in AD leads to accelerated clearance and lower level [146]. Much more should be still studied in order to better understand the mechanism underlying the TTR protection in AD. The strategy of using TTR stabilization as a therapeutic target in AD needs to be accurately evaluated taking into account that TTR is decreased in CSF and in sera of AD patients [147] and also considering that TTR monomers seem to bind more Aβ than do tetramers [125]. 

Generally, inhibition of protein-protein interactions is challenging because it requires the modulation of typically large, relatively flexible surface area [148]. This is normally the reason why small molecules often lack selectivity [149]. Monoclonal antibodies and other protein therapeutics have the advantage to be selective, but they suffer from poor oral bioavailability, high cost and susceptibility to proteolysis [150]. All these disadvantages pushed researchers to study peptides and peptidomimetics as promising therapeutics in the field of protein-protein interactions, because they can afford selectivity and affinity, thanks to their size in midway between small-molecules and protein therapeutics [148]. Their relatively cheap and modulable chemical synthesis offers the opportunity to incorporate also elements enhancing bioavailability and stability. Finally, peptidomimetic foldamers give the possibility to mimic the secondary structure of the peptide sequence, generally involved in the interaction [151]. Understanding protein/peptide self-assembly using structural and biophysical chemistry continues to offer the possibility to investigate the binding epitopes involved in the interaction and to provide guidance for future development of therapeutics. 

**Figure 4:**
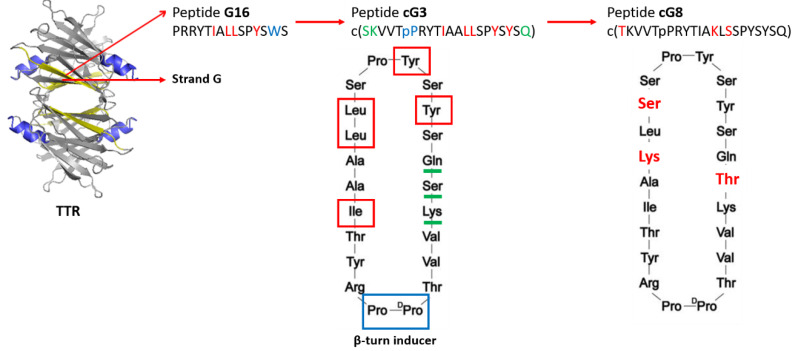
Schematic representation of the design development of peptide cG8, as first TTR-derived peptide inhibitor.

Aβ binds to TTR through two different binding domains: strand G in the inner β-sheet (residues 102–117), and the EF helix/loop (residues 74–83). The first example of peptides that mimics Aβ-binding domains of TTR was reported by Murphy et al. in 2014 (Figure 4) [152]. Through a structure-activity relationship study, they identified, for strand G, important features required for binding to TTR: the need of a minimum length of 10 residues, the importance of the hydrophobic hexamer TIAALL as well as *C*-terminal residues SPYS or SPYSYS, the relevance of hydrophobic residues isoleucine and leucine in the *N*-terminal domain (I107, L110, L111) and aromatic groups in the *C*-terminal domain (Y114, Y116). They identified a linear peptide (G16) able to bind Aβ and reduce its toxicity in a dose-dependent manner, even if it increased the average size of Aβ aggregates, unlike wild-type TTR [152] (Figure 4). Because G16 was less effective than the parent TTR at protecting neurons from Aβ toxicity, it was thought that this was imputable to a lack of β-strand/loop/β-strand structure, typical of the Aβ-binding domain. To cope with that, the peptide sequence has been transplanted onto a β-hairpin template by the introduction of a β-turn inducer (DPro-LPro) and an N-to-C cyclization to further restrict conformational restriction. The imposition of structural constraints generated a much improved peptidomimetic of the Aβ binding epitope on TTR (cG3, Figure 3) [153]. Successively, additional changes had the aim to improve the solubility, specificity and stability of the Aβ-inhibitor. Compound cG3 showed a better activity compared to G16 but it was not as effective as the wild-type TTR. The explanation was probably related to its still not enough stabilized antiparallel β-strand structure and its tendency to self-aggregate. Improvements concerning the β-sheet tendency and hydrophobicity were explored by TANGO algorithm which helped to identify specific mutation on the cyclic peptide sequence able to retain or stabilize the conformational structure while minimizing the self-association. This approach allowed to identify cG8 (Figure 4), a cyclic peptide which demonstrated in multiple complementary techniques to cluster Aβ into large weakly associated aggregates, thus blocking Aβ in a non-fibrillar aggregation stage and accelerating the Aβ clearance by natural mechanisms [154]. 

In a study comparing protein versus peptide [155], each designed as a mimic of the Aβ-binding domain on wild-type TTR, both mTTR (engineered protein) and cG8 (cyclic peptide) resulted effective at inhibiting amyloid formation by either Aβ isoform, Aβ 1-40 and Aβ 1-42. The results obtained by ThT fluorescence spectroscopy showed that mTTR and cG8 are not broad-spectrum anti-amyloid agents, because they recognize similar epitopes that Aβ and amylin share but that α-synuclein does not possess. Nevertheless, mTTR has the advantage to be more effective to lower concentration, having a strong impact on both the morphology and the quantity of Aβ deposits on cell, while cG8, thanks to its smaller size, results in better stability against proteolysis and less interferences from nonspecific biological materials. It is hypothesized that the greater efficacy of mTTR is attributable to a relative stable anti-parallel two β-strand conformation that fully mimics TTR’s Aβ binding site, while cG8 shows a conformational heterogeneity [155]. These findings highlight the fact that the design of TTR-derived anti-Aβ agents requires a correct balance between advantages and disadvantages of using a protein versus peptide as therapeutic, and a compromise between efficacy, specificity, stability and conformational behavior is demanding. This consideration opens the way to the use of peptidomimetic foldamers, for example, as a new approach which might resolve a major issue in the use of peptides as drugs, by stabilizing secondary conformations similar to natural peptides and retaining the selectivity due to the lateral chains [156].

### 3.3. Cystatin C (CysC)

Human cystatin C (CysC), a protein encoded by the CST3 gene, is a member of cystatin 2 family. CysC is the most spread cystatin in human body fluids, secreted by all nucleated cells and it is a natural inhibitor of papain-like and legumain-like cysteine protease [157]. CysC is a basic protein composed by 120 amino acid residues (13.3 kDa), characterized by three main domains interacting with the target enzymes: the *N*-terminal disordered segment (S1-V10) and the two hairpin loops L1 (^55^QIVAG^59^) and L2 (^105^PWQG^108^). Under physiological condition, CysC is a monomeric protein. In healthy people, the CysC concentration in the CSF is six times higher than that of blood plasma [158], as a result of a large expression of this protein by the brain tissue (neurons, astrocytes, endothelial, and microglial cells) [159]. The principal physiological role of CysC is the inhibition of cathepsins B, H, K, L and S which are acidic proteinases, lysosome-located, involved in the protein turnover and in the processing of neuropeptides in the CNS. These cathepsins are studied in AD because it has been observed that cathepsin-immunoreactive material is associated with senile plaques and neurofibrillary tangles [160]. Furthermore, CysC itself is a target for proteases and its function is inactivated by cathepsin D and elastase [161]. 

**Figure 5:**
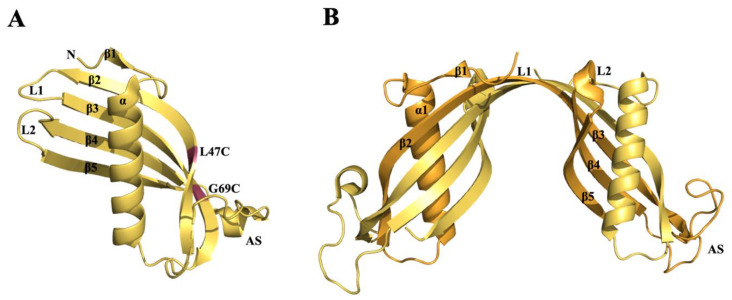
Graphic representation of two CysC X-ray crystal structures. (**A**) Cartoon of CysC-stab1 (PDB code: 3GAX) folded as a monomer. N = *N*-terminus, L1 = loop1, L2 = loop2, AS = irregular appending structure. The CysC-stab1 mutations L47C and G69C are colored in red. (**B**) Cartoon representation of CysC (PDB code: IR4C) folded as 3D domain-swapped dimer. The two molecules, which compose the dimer, are colored in yellow-orange and bright orange. L2 is conserved while L1 is transformed to form the dimer.

In vitro experiments showed that a slight change in pH or temperature and the addition of small amount of denaturing agents induce the dimerization and oligomerization of wild-type CysC [162,163]. CysC itself tends to form amyloid fibril and to precipitate with other amyloidogenic proteins such as APP full-length, secreted APPα and its processing products Aβ peptides (Aβ1-40, Aβ 1-42) [164]. A single point mutation of CysC (Leu68Gln) leads to hereditary cystatin C amyloid angiopathy (HCCAA) [165]. The fibril formation pathway is analogue to that suggested for other amyloidogenic proteins: a single point mutation is responsible of a conformational change which leads to expose hydrophobic surfaces promoting the self-association and thus the fibril deposition [166,167]. In 2010, the crystal structure of human CysC-stab1 mutant (L47C and G69C) was solved [168]. For the first time, it was proved that human CysC folded as monomeric protein with a canonical cystatin structure characterized by a long α-helix running across a five-stranded antiparallel β-sheet stabilized by two hairpin loops, L1 and L2 [168] (Figure 5A). Until then, all the crystallization experiments had led to obtain the dimeric form of CysC as a result of a 3D domain swapping, the same structural arrangement firstly observed in diphtheria toxin [169,170] (Figure 5B). The three-dimensional domain swapping consists in a mechanism by which CysC conserves its monomeric secondary structure, but the protein is refolded as a 2-fold symmetric dimer (Figure 5). The dimer is structured through the exchange of three-dimensional ‘subdomains’ between the two monomers. Some studies suggested that the swapping dimerization could be the mechanism by which CysC forms oligomers and fibrils [163,171,172], moreover the relationship between the CysC swapping dimerization and its fibrillization has not been clarified yet. 

Several studies reported that CysC plays a protective role against several pathological manifestations such as tumor metastasis, inflammation, viral and bacterial infections and neurodegenerative disorders [166]. Moreover, the variation of CysC levels, in specific tissues or body fluid, might be used as diagnostic marker to study the onset or progression of various diseases. In 2016, Mathews and Levy have summarized, in an exhaustive review, the changes in CysC expression or function related to several CNS aging-dependent diseases [173]. Several studies discussed the potential and the controversial role of CysC in AD pathogenesis. 

The co-localization of CysC and Aβ has been observed in the cerebral arteries of patients affected by cerebral amyloid angiopathy (CAA) [174], in parenchymal and vascular amyloid deposits in brains of patients with Alzheimer disease [175] and in sporadic inclusion-body myositis (sIBM) muscle fibers [176]. All these evidences strengthen the hypothesis that CysC might play an important role even in AD. Controversial results have been obtained, investigating the connection between the polymorphism CST3 gene, encoding for CysC, and AD developing. In a studied published in 2008, it was reported that, in Mainland Chinese patients and the healthy controls no statistical difference exists between CST3 genotype and allele frequencies [177]. In addition, in 2012 another research group investigated the possible association between CST3 G73A polymorphism and AD. The result showed that the CST3 G73A polymorphism is associated with AD in Caucasian populations, but not in Asians [178]. Instead, a synergic correlation has been demonstrated between the CST3 polymorphism and apolipoprotein E (APOE) ε4 alleles. Experimental data suggested a synergistic association among the CST3-A allele, APOE4 and AD in elderly AD patients [179,180]. 

The Aβ peptide accumulation has a key role in AD pathogenesis. A strategy to decease the cerebral Aβ level is to activate the endogenous pathways inducing Aβ degradation and scavenging. Cathepsin B (CatB), one of the enzymes implicated in Aβ degradation, cleaves the *C*-terminal of Aβ1-42 peptides decreasing the Aβ levels [181]. It was hypothesized that the reduction of CysC, endogenous inhibitor of CatB, can reduces Aβ levels. A study reported that the genetic ablation of CysC, in transgenic mice overexpressing human amyloid precursor protein (hAPP) with familial AD (FAD)-linked in Swedish and Indiana mutations (hAPP-J20 mice), increases CatB activity in the brain and drastically decreases Aβ levels [182]. This protective effect is lost in hAPP mice without CatB. The majority of AD patients do not possess the FAD mutation, so the same experiment was carried out on hAPP wild type (hAPPWT) showing that CysC-CatB affects Aβ levels in hAPPWT mice in a similar manner as in hAPPFAD mice [183].

Conversely, other studies have reported neuroprotective effects of an increased CysC expression in animal models. It was reported that transgenic mice expressing human higher CysC levels than normal displayed a drastic decrease in Aβ fibril deposition [184]. Moreover, CysC overexpression showed to reduce the AD plaque formation in hAPP-transgenic mice [185]. In another study the CatB-deficient mice were analyzed and the CysC overexpression decreased the total amyloid plaque deposit [182]. 

In summary, CysC showed a controversial role in AD: on one side it seems to regulate the Aβ levels directly binding to Aβ and inhibiting its aggregation, on the other its being a substrate for protease CatB seems to be competitive for Aβ degradation.

#### β-Amyloid-Binding Sites on CysC

**Figure 6:**
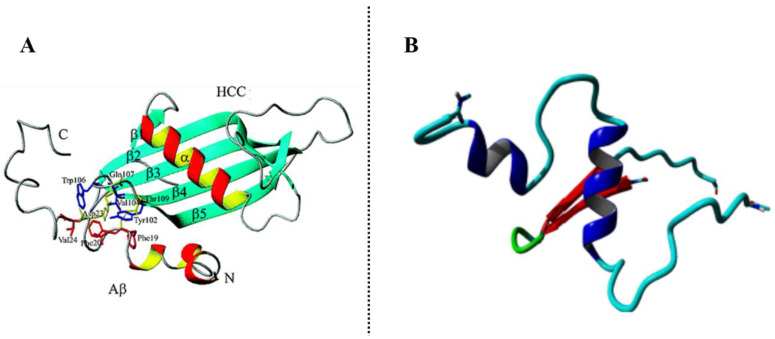
(**A**) Interaction structure of the CysC-Aβ complex revealed by molecular dynamics simulation in the study of Przybylski et al. [186] (**B**) The binding complex from the docking output between Aβ and CysC fragment in the study of Sharma et al. [188].

Sastre et al. were the first to show that the association of CysC with Aβ causes an inhibition of fibril formation. During their ELISA affinity assay, they found that the monoclonal antibody 6E10, which binds to residues 1–17 of Aβ, abolished the CysC binding to Aβ-coated plates, thus suggesting that the binding site within Aβ is within the amino-terminal domain of the peptide [164]. Successively, proteolytic excision mass spectrometry analyses, conducted by Przybylski et al. [186], revealed that the CysC binding site is in the central region of Aβ within residues 17–28 which is critically important for the Aβ structure and aggregation. This sequence contains the hydrophobic core of the Aβ peptide (LVFFA) and the β-turn for fibril formation located within residues 25–28. This region of Aβ interacts with the *C*-terminal β-hairpin motif of CysC within the L2 loop and β5 strand comprising residues 101–117. A structure model of CysC-Aβ complex (Figure 6A) obtained by molecular docking simulation showed that, while the initial Aβ structure changed during the simulation and did not have a large influence on the structure and stability of the complex, CysC structure (residues 101–117) was kept stable and seems to have the major impact on the hydrophobic and electrostatic interactions. Residues Tyr-102, Val-104 and Trp-116 interact with Phe-19, Phe-20 and Val-24 on Aβ peptide, while Gln-107 and Thy-109 establish hydrogen bonds with the carbonyl group of Phe-19 and Asp-23 [186]. The same research group characterized structures and affinities of both Aβ and CysC not only by enzyme-linked immunosorbent assay-like assay, surface plasmon resonance and nano-ESI-FTICR-MS but also by making Ala-scan analysis of CysC 101–117 fragment in order to study the importance of each residue in the interaction binding. By this latter, they found that the substitution of the previous important residues discovered by simulation by an Ala did not decrease or abolish the Aβ-CysC complex. Rather residues Gln-107, Gly-108, Ser-113, Lys-114 and Ser-115 showed to be more involved in the complex stabilization. Deletion of the *C*-terminal amino acids in the CysC 101-117 fragment resulted to affect strongly the affinity and revealed the need not only of hydrophobic but also electrostatic interactions in the formation of the complex. Structural studies by circular dichroism and NMR conducted on CysC 101–117 fragment demonstrated the absence of a well-defined structure with a weak tendency to bend in the middle part of the sequence [187]. The Aβ-binding CysC sequence could be the basis for the design of potential inhibitors of amyloid β-aggregation process but much more attention should be taken on the conformational requirements for CysC-epitope binding to Aβ. 

Recently, all-atom molecular dynamic simulations and rigid body protein-protein docking underlined the important roles of hydrogen bonding and hydrophobic interactions in the stability of the complexes between Aβ and CysC and thus the importance of noncovalent forces in biomolecular interactions of therapeutic significance. During all the simulation, Aβ explores different conformational rearrangements with a major secondary structure element being an α-helix, contrary to CysC whose secondary structures revealed a relative rigidity with a preserved β-sheet as representative structure (Figure 6B) [188]. These latter findings showed that the possible mechanism of the CysC β-hairpin domain might be a stabilization of an α-helix intermediate conformation of Aβ which might contribute to its monomer-state stabilization and so to its metabolic degradation. 

The first example of CysC-derived Aβ aggregation inhibitors have been showed by Przybylski et al. [186] Using an in vitro assay of Aβ aggregation, they found that CysC 101–117 peptide was able to reduce the formation of Aβ aggregates with a time- and concentration-dependent inhibitory effect [186]. More recently, two CysC fragments have been found to play the role of a steric zipper motif which could enhance the conformational change of CysC and very easily form complementary β-sheet structures, involved during the formation of amyloid deposits: the loop L1 region and the *C*-terminal fragment. Particularly, Ala-52 to Asp-65 fragment has been proved to have high fibrillization propensity and potentially to be able to form a steric zipper. In the protein structure (3GAX), this fragment is located in the first hairpin and consists of sequences of β-strands 2 and 3 and the loop L1 which connects these strands [189]. At the moment, nothing is known about the implication of the *C*-terminal fragment on the amyloid behavior of CysC but much more should be studied about this fragment because of its characteristic β-harpin conformation and the fulfilling conditions for being an effective steric zipper, probably the one that can recognize the α-helix intermediate conformation of Aβ.

### 3.4. Apolipoprotein A1 (ApoA1)

Apolipoprotein A1 (ApoA1) is the main component of high-density lipoprotein (HDL), playing an important role in lipid transport, constitution and metabolism of HDL cholesterol [190,191,192,193]. It is a plasma protein composed of 243 amino acids, encoded by exons 3 and 4 of the APOA1 gene, with a global weight of 28 kDa [194]. Human ApoA-1 is synthesized as preproapoA-1, a 267 amino acid precursor apolipoprotein, which undergoes intracellular co-translational proteolytic cleavage into proapoA-1 and successively proapoA-1 is cleaved to mature plasma ApoA1 in human plasma [195,196]. Its mature form is essentially expressed in the small intestine and in the liver [190,197]. About 95% of the protein is bound to mature HDL but a few ApoA1 circulates in a lipid-free form [198].

In vivo, ApoA1 has been identified as an amyloidogenic protein among other apolipoproteins [199]. The *N*-terminal fragment, essential for the stabilization of the secondary structure of ApoA1 [200], is generally highly conserved in apolipoprotein-derived amyloidosis and seems to have an important role in the formation of amyloid fibrils [190,201,202,203,204], in particular in those amyloidosis affecting patients with chronic inflammatory disorders (secondary or reactive amyloidosis) [205]. The *C*-terminal domain is the minimal lipid-associating domain of ApoA1 and allows binding to lipids with high affinity [206,207]. Lysine and arginine residues in this region are responsible for this strong affinity because they can bury into the membrane the hydrophobic part of their side chains [208,209]. While the *C*-terminal fragment is anchored into the membrane and therefore difficult to access, the *N*-terminus, on the other side, results to be more accessible for the interactions with other possible components.

**Figure 7:**
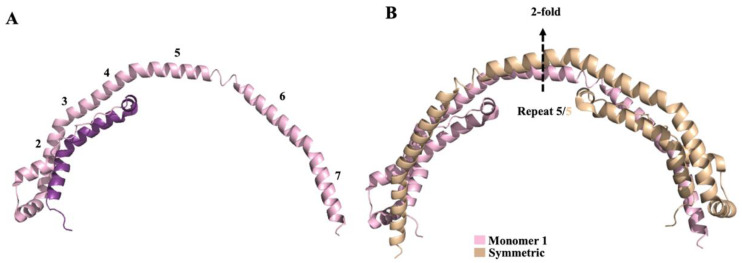
Graphic representation of Δ(185–243)apoA-I crystal structure (PDB code 3R2P). (**A**) Monomer of Δ(185–243)apoA-I. *N*-terminus 3-43 residues are colored in violet, 44-184 ligthpink. Helix repeats 1-7 are reported. (**B**) Homodimer representation of Δ(185–243)apoA-I. The crystallographic 2-fold axis crossed the middle of sequence repeat 5.

In plasma, as previously mentioned, ApoA1 circulates in a lipid-free, lipid-poor, and lipid-bound form, therefore it has a flexible and adaptable structure. The adaptable nature of ApoA1 hampered high resolution structural studies. To date, two different human ApoA1 truncate structures are present in the PDB data bank (PDB code: 1AV1 [200] and 3R2P [210]). The first ApoA1 crystal structure, deposed in 1997 (code 1AV1), corresponds to a Δ(1–43) truncated mutant of human ApoA1. Due to the low resolution (4 Å) no detailed structural information can be extrapolated. Although, four-helical horseshoe-shaped molecules, assembled in the crystal to form a tightly associated elliptical ring, are visible. This crystal structure did not furnish any data about the *N*-terminal 43 residues [200]. In a study published in 2011, the structure of native Δ(185–243)ApoA1 (code 3R2P, resolution at 2.2– Å) was obtained, thus giving the information about the *N*-terminal residues (residues 3–43). One molecule of Δ(185–243)ApoA1 is composed by 80% of helix and forms roughly a half-circle (Figure 7A). Each monomer generates a homodimer interacting with its symmetry-related molecule with a semi-circle architecture (Figure 7B) [210].

ApoA1-induced amyloidosis often trigger asymptomatic hepatopathy and nephropathy [211]. The hereditary one, the most frequent form, involves mutants of ApoA1 responsible of a systemic amyloidosis [212,213]. Besides this hereditary form, ApoA1 amyloidosis can be found as a non-hereditary form, characterized by the wild-type protein deposition [214]. 

So far, among the 50 ApoA1 variants described [193], about half of them are known to be associated with a decreased plasma level of HDL-ApoA1. These ApoA1 variants have undeniable interest because they may affect lecithin-cholesterol acyltransferase (LCAT) activity and promote the formation of amyloidosis [190]. Indeed, patients presenting mutations of the ApoA1 gene are more at risk of developing ApoA1 hereditary amyloidosis [215]. These mutations are clustered in 2 principal regions of the protein: residues 26–90 in the *N*-terminal part and residues 154–178. Hereditary ApoA-I amyloidosis is characterized by deposition of the *N*-terminal 80‒100-residue fragments as amyloid fibrils in peripheral organs. Mutation seems to perturb the native protein structure, making it more susceptible to proteolysis and thereby to the release of the *N*-terminal amyloidogenic fragment [216,217]. 

The G26R mutation has been associated to hereditary amyloidosis leading to renal failure [213,218]. Recently, Mizuguchi et al. studied the role of the *N*-terminus (1‒83) of this variant in the onset of amyloidogenesis. Using ThT method and atomic force microscopy, they showed that the fragment 14-22 is essential for the fibril formation, while fragments 32–40 and 50–58 have a role in the nucleation process. Using circular dichroism, they also showed that the fragment 14–22 allows β-transition and fibrilization [216]. Studies with electron paramagnetic resonance spectroscopy [219], X-ray crystallographic studies [220] and hydrogen-deuterium exchange mass spectroscopy [221] showed that the G26R mutation induces helix destabilization of the protein in the *N*-terminal domain leading to a transition of residues 14‒58 to a β-sheet conformation (Figure 7) [216]. In mature amyloid fibrils, ApoA1 *N*-terminal fragments are assembled in a parallel, in-register β-sheet structure and the protofilaments of ApoA1 present a β-strand-loop-β-strand structure [205,217].

Immunohistochemical studies revealed the presence of ApoA1 in senile plaque [222], suggesting a potential cross-interaction with amyloid β peptides. Indeed, Koudinov et al. demonstrated that ApoA1-containing HDL particles can bind to circulating Aβ peptide, as revealed by SDS PAGE and immunoblot analysis [223,224]. Notably, a correlation between the decreased levels of plasmatic ApoA1 and the occurrence and severity of AD has been showed [225]. 

Vollbach et al. studied the impact of different polymorphisms of ApoA1 on AD, in particular in the promoter region of ApoA1, the sequence of DNA initiating transcription. Some presumed effects of polymorphism in this region impact serum levels of ApoA1 or the function of the protein. These polymorphisms are then shown to be involved in the physiopathology of AD. For example, the G/A substitution at position 75 pb is implicated in an elevated risk for AD [226]. 

A lot of naturally occurring ApoA1 variants have so far been identified, impacting levels of HDL and amyloidosis of the protein. ApoA-1-Milano (ApoA1M) was the first natural variant of ApoA1 identified, with a cysteine replacing an arginine in the 173 position [209,227,228,229]. ApoA1 and ApoA1M have been shown to be both able to prevent the cytotoxicity induced by Aβ1-42 in brain endothelial cells [230]. In transgenic mice, the chronic intravenous treatment with ApoA1M resulted in a decrease the level of cerebral soluble, insoluble and membrane-bound forms of Aβ1-42 and Aβ1-40 [230]. 

ApoA1 has a positive effect on Aβ, preventing fibril formation and attenuating Aβ toxicity [231]. The binding of ApoA1 to Aβ1-40 have been shown to contribute to maintain Aβ in solution, thus preventing its deposit within the brain in some pathological conditions. ApoA1 levels are also found to be significantly lower in AD patients compared to controls [223] whereas high levels of ApoA1 are associated in lowering risks of dementia [232]. There might be an important correlation between Alzheimer’s disease and a decrease of ApoA1 levels in plasma [233]. Besides, men with high levels of LDL cholesterol, meaning low levels of HDL and ApoA1, are more at risk to develop AD [234].

An increased dissemination of Aβ1-42 deposition has been observed in KO ApoA1 and KO ABCA1 mice, with ABCA1 as an ATP binding cassette regulating the cholesterol efflux from cells to ApoA1. Moreover, an increase of plasma levels of Aβ1-42 and an aggravation of memory deterioration impacting negatively dendrite architecture have been also identified in KO ApoA1/ApoE mice, suggesting an important role of more than one apolipoprotein in the Aβ1-42 clearance [235].

By using a blood-brain barrier (BBB) model, an increased Aβ efflux from the basolateral side of the BBB has been shown when ApoA1 is in a discoidal HDL form. On the contrary, there is no effect on the efflux when ApoA1 is in a spheroidal HDL or in the plasma pool. ApoA1 in a discoidal HDL can cross BBB and reduce fibrils amount and extension by remodeling Aβ fibrils [236].

Conversely, it should be notice that ApoA1 might have an indirect role in the pathogenesis of AD. In fact, a study using AD mouse models showed that cognitive deficits in memory and learning could be limited by circulating ApoA1 overexpression despite the concomitant deposition of Aβ plaques. These results seem to suggest an indirect role of ApoA1, which would rather reduce neuroinflammation and cerebral amyloid angiopathy than directly bind to Aβ [237].

ApoA1 is assumed to be an amyloid protein able to decrease the Aβ fibrilization by affecting in vitro the morphology of the fibrils [23,231]. ApoA1 prevents the formation of high molecular weight aggregates of Aβ1-42 and decreases Aβ1-42 toxicity in primary brain cells. The inhibition of Aβ1-42 aggregation is ApoA1 concentration-dependent [238]. 

Furthermore, by in vitro assays (ThT fluorescence spectroscopy, SDS-PAGE and immunoblot), Radosveta et al. found ApoA1 to have a strong affinity for the amyloid precursor protein (APP) and for Aβ1-40 (Kd = 6 nM) and to be able to inhibit the β-sheet formation and the Aβ-induced cytotoxicity (IC50 = 580 nM) [231]. ApoA1 interferes with Aβ-induced lipid peroxidation and, in the presence of ApoA1, Aβ aggregates are less neurotoxic than pure Aβ fibrils [201].

All this evidence supports a beneficial role of ApoA1 on Aβ aggregation and toxicity, thanks to a direct interaction and the formation of a complex between ApoA1 and Aβ. However, the mechanism of this protective effect has not been yet clearly elucidated. It is evident that it might be interesting to study this cross interaction with Aβ1-42. Although the crystallographic structure of ApoA1 has already been investigated [200], the crystal information of the Aβ-ApoA1 complex is still missing. 

Considering the interesting properties of ApoA1 on Aβ regulation, the next step would be deeply exploring the ApoA1-Aβ complex and the corresponding binding epitopes, in order to design peptides mimicking this interaction, as observed for TTR and CysC. Therapeutic approaches inspired by ApoA1 have been developed primarily to increase levels of ApoA1 in order to treat atherosclerosis and acute coronary syndrome. Among them we can count HDL infusion and mimetics, recombinant LCAT, ApoA1M infusions and ApoA1 transcriptional upregulators [239,240,241]. Now, few of them have been explored to study a possible correlation between the increased level of ApoA1 and the improvement of dementia in AD patients. 

The only exception is represented by the ApoA1 transcriptional up-regulator RVX-208, developed by Resverlogi. It has been demonstrated that this compound leads to an increase of circulating levels of ApoA1 [242,243]. Interestingly, RVX-208 has been inserted into phase 1a clinical trial for the treatment of AD and showed the ability to increase the Aβ1-40 efflux from the brain [244].

Furthermore, reconstituted HDL (ApoA1 + soy phosphatidylcholine) [245] were tested on mice to study if they could lower plasma levels of Aβ. In treated mice a reduction of soluble Aβ1-42 and Aβ1-40 within 24h has been observed but no effects were observed on Aβ and on inflammation after chronic treatment [246]. 

As mentioned above, ApoA1 can modulate Aβ aggregation and neurotoxicity and the Aβ-binding domain in ApoA1 might constitute a novel framework for the design of inhibitors of Aβ toxicity. 

Thus, the interest in the identification of homologous peptides of the *N*-terminal Aβ1-42 binding domain is growing. The sequence 42-GNLLTLD-48 has been identified as a homologous sequence present in the *N*-terminal part in many mammalians’ apolipoproteins. The sequence 42-LNLKLLD-48 is the corresponding sequence in Homo sapiens. The results of ThT binding assays showed that an incubation of this heptapeptide with Aβ blocked the formation of Aβ/ApoA1 complexes, confirming that this sequence in the *N*-terminus of ApoA1 might be the binding site for Aβ [201].

Another possible approach for the treatment of AD could be increasing the levels of HDL, thus consequently the levels of ApoA1. Analogs of the amphipathic α-helical structure of ApoA1 have been already developed for other therapeutic purposes. These peptides have an impact on metabolism and biological activities of HDL [243]. The 18-amino-acids long peptide DWLKAFYDKVAEKLKEAF (18A) was designed to mimic an ApoA1 α-helix and was found to associate with liposomes and to displace ApoA1 from HDL [207,208,209]. Clinical studies investigating these kinds of peptides have been performed on patients with coronary heart disease [247]. Thus, increasing plasma levels of ApoA1/HDL would be a new interesting strategy for the improvement of the cognitive function in Alzheimer’s disease, although a direct evidence of this is still missing.

## 4. Conclusions

In this review, we highlighted the therapeutic potentiality of Aβ1-42 cross-interactions with other amyloid proteins. Among the several amyloid proteins interacting with Aβ, we chose four of them which in literature have been considered the most interesting for developing new therapeutic approaches for AD. We showed that Aβ/Tau amyloid cross-interactions likely contribute to the synaptic dysfunction involved in AD, but much more must be still performed to better understand the role and influence that each single protein has on the other. We advised that future inhibitors should target common interface region of Aβ and Tau and the determination of the high-resolution structure of Aβ-Tau complex would contribute to the understanding of the key binding residues for optimizing the inhibition of amyloid seeding in AD.

Furthermore, we illustrated the three different strategies which have been employed to enhance or mimic the TTR-Aβ interaction in order to improve the clearance of Aβ peptide and consequently avoid its aggregation into amyloid aggregates. This part was the occasion to underline the great challenge required for modulate protein-protein interactions and the important role of peptides and peptidomimetics as promising therapeutics in the field of the cross-interactions, because they can afford selectivity and affinity and, especially for peptidomimetic foldamers, they give the possibility to mimic the secondary structures, generally involved in the interaction.

Even the Aβ-binding CysC sequence could be the basis for the design of potential inhibitors of amyloid β-aggregation process. In this review we could highlight a possible mechanism by which a CysC β-hairpin domain might be stabilize an α-helix intermediate conformation of Aβ, thus contributing to its monomer-state stabilization and so to its metabolic degradation. Finally, evidences support a beneficial role of ApoA1 on Aβ aggregation and toxicity, thanks to a direct interaction and the formation of a complex between ApoA1 and Aβ. However, the mechanism of this protective effect has not been yet clearly elucidated. It is evident that it might be interesting to study this cross interaction with Aβ1-42 and the exploration of the Aβ-binding domain in ApoA1 might constitute a novel framework for the design of inhibitors of Aβ toxicity.

The amyloid cross-interactions seem to have a positive effect on the stabilization of the native state and destabilization of incorrectly folded state of amyloids. Thus, by taking inspiration from the heterobifunctional PROTAC approach [248,249] and with the purpose of boosting the positive interaction between two amyloid proteins, two covalently linked protein-binding molecules or peptidomimetics can be designed in this type of protein-protein interaction and exploit as a new therapeutic strategy. The formation of a stable ternary complex between the two amyloids, close together through the PROTAC construct, should improve the approach of the two proteins and allow the natural positive effect of the cross-interaction.

In conclusion, cross-interactions between Aβ and other amyloid proteins have been shown to concern potentially therapeutic interventions against AD. This review allowed to emphasize the role of the cross-interactions in the modulation of AD but also to open the idea that cross-interactions might also modulate amyloidosis in other pathologies.
